# Expression and regulation of drug transporters in vertebrate neutrophils

**DOI:** 10.1038/s41598-017-04785-4

**Published:** 2017-07-10

**Authors:** Matthew J. Foulkes, Katherine M. Henry, Julien Rougeot, Edward Hooper-Greenhill, Catherine A. Loynes, Phil Jeffrey, Angeleen Fleming, Caroline O. Savage, Annemarie H. Meijer, Simon Jones, Stephen A. Renshaw

**Affiliations:** 1 The Bateson Centre, The University of Sheffield, Sheffield, S10 2TN UK; 2 Department of Chemistry, The University of Sheffield, Sheffield, S3 7HF UK; 3 Department of Infection, Immunity & Cardiovascular Disease, The University of Sheffield, Sheffield, S10 2RX UK; 4 Institute of Biology, University of Leiden, 2333 CC Leiden, The Netherlands; 5 Immuno-Inflammation Therapy Area Unit, GlaxoSmithKline Research and Development Ltd., Stevenage, SG1 2NY UK; 6 Rare Diseases Research Unit, Pfizer Ltd., Cambridge, CB21 6GP UK; 7 Department of Physiology, Development and Neuroscience, University of Cambridge, Cambridge, CB2 3EG UK; 8 Department of Medical Genetics, University of Cambridge, Cambridge Institute for Medical Research, Addenbrooke’s Hospital, Cambridge, CB2 2XY UK

## Abstract

There remains a need to identify novel pro-resolution drugs for treatment of inflammatory disease. To date, there are no neutrophil-specific anti-inflammatory treatments in clinical use, perhaps due to our lack of understanding of how drugs access this complex cell type. Here we present the first comprehensive description and expression of both major classes of drug transporters, SLC and ABC, in resting human blood neutrophils. Moreover, we have studied the expression of these carriers in the tractable model system, the zebrafish (*Danio rerio*), additionally examining the evolutionary relationship between drug transporters in zebrafish and humans. We anticipate that this will be a valuable resource to the field of inflammation biology and will be an important asset in future anti-inflammatory drug design.

## Introduction

New treatment approaches are urgently needed for inflammatory diseases in which inflammation fails to resolve spontaneously, for example Chronic Obstructive Pulmonary Disease (COPD). Key innate immune cells, particularly neutrophils, are recruited to the affected organs in response to tissue damage or infection and may be retained there by proinflammatory signalling. These unwanted neutrophils can contribute to tissue damage and their removal is an important aim of anti-inflammatory therapy^1^. To date, there are no neutrophil-specific anti-inflammatory treatments in clinical use. Barriers to targeting this key cell include our lack of understanding of drug delivery into neutrophils and a lack of drug-screening assays with physiological readouts.

Zebrafish (*Danio rerio*) are an especially useful model organism in which to study neutrophil behaviour^2^, allowing genetic manipulation and subsequent *in vivo* imaging of the inflammatory process. The small size and large numbers of larvae produced in a single mating allow drug libraries to be screened efficiently. Drug screening approaches are routinely used by zebrafish groups around the world and have resulted in the identification of several neutrophil-targeted compounds^3–6^.

Knowledge of the mechanisms by which drug-like molecules enter neutrophils is important for the design of pharmaceuticals to modulate neutrophil function, both for improved drug screening strategies and for improved clinical outcome. Within the last twenty years, it has become clear that drug transporters (proteins that transport exogenous compounds across the cellular membrane) are crucial for absorption, distribution and excretion of exogenous compounds^7, 8^. No study has examined drug transporter expression in either neutrophils or zebrafish to date.

Drug transporters comprise two main families: solute carrier (SLC) and ATP-binding cassette (ABC) transporters. In humans, there are 396 members of the SLC family and 51 members of the ABC family, according to the HUGO Gene Nomenclature Committee (HGNC) database (April 2015)^9^, and these exist for the transport of metabolites and ions into and out of cells^10^. Indeed, the metabolite-likeness of many drugs in clinical use is highly supportive of the role of these transporters in the movement of therapeutic compounds across cell membranes^11^.

SLC transporters are considered responsible for the influx of molecules into the cell. In the same way, as one drug may act upon many target proteins, one drug may be transported by multiple transporters, thus making the identification of the transporters responsible for the entry of each compound challenging. The clearest example of drug transport by a SLC protein is perhaps in the treatment of pancreatic cancer with gemcitabine, which is predominantly transported by the SLC29A1 transporter (previously known as ENT1)^12, 13^.

ABC transporters are generally considered to be responsible for efflux of compounds from the cell, and first came to the attention of the pharmacokinetics field when it was noted that in patients carrying particular mutations in ABC proteins, some drugs presented toxicity issues^14, 15^. Clearly, it is important to consider both influx and efflux of potentially therapeutic compounds during drug discovery and development.

Despite drug transporter expression being determined in humans and other animal models^16, 17^, to our surprise, we were unable to identify either a published analysis of drug penetration into neutrophils or a description of drug transporter expression in neutrophils. In our hands, predicting efficacy and effective doses of drugs against neutrophils has been difficult both *in vitro* and *in vivo* ^4, 18–20^. We therefore hypothesised that drug entry into neutrophils might be dependent on the differential expression and regulation of drug transporters, with important consequences for screening methodologies and rational anti-inflammatory drug design.

Here we present, to the best of our knowledge, the first comprehensive analysis of expression data of the SLC and ABC drug transporter families in both human and zebrafish neutrophils. Our analysis has also allowed for the identification of several previously unannotated SLC and ABC paralogues in zebrafish. These data are an important advance in both the neutrophil and zebrafish fields and may be useful in designing novel successful compounds for drug screening experiments and, ultimately, the treatment of inflammatory disease.

## Results

### Distinct subsets of transporter proteins are expressed and regulated in primary human neutrophils

**Figure 1:**
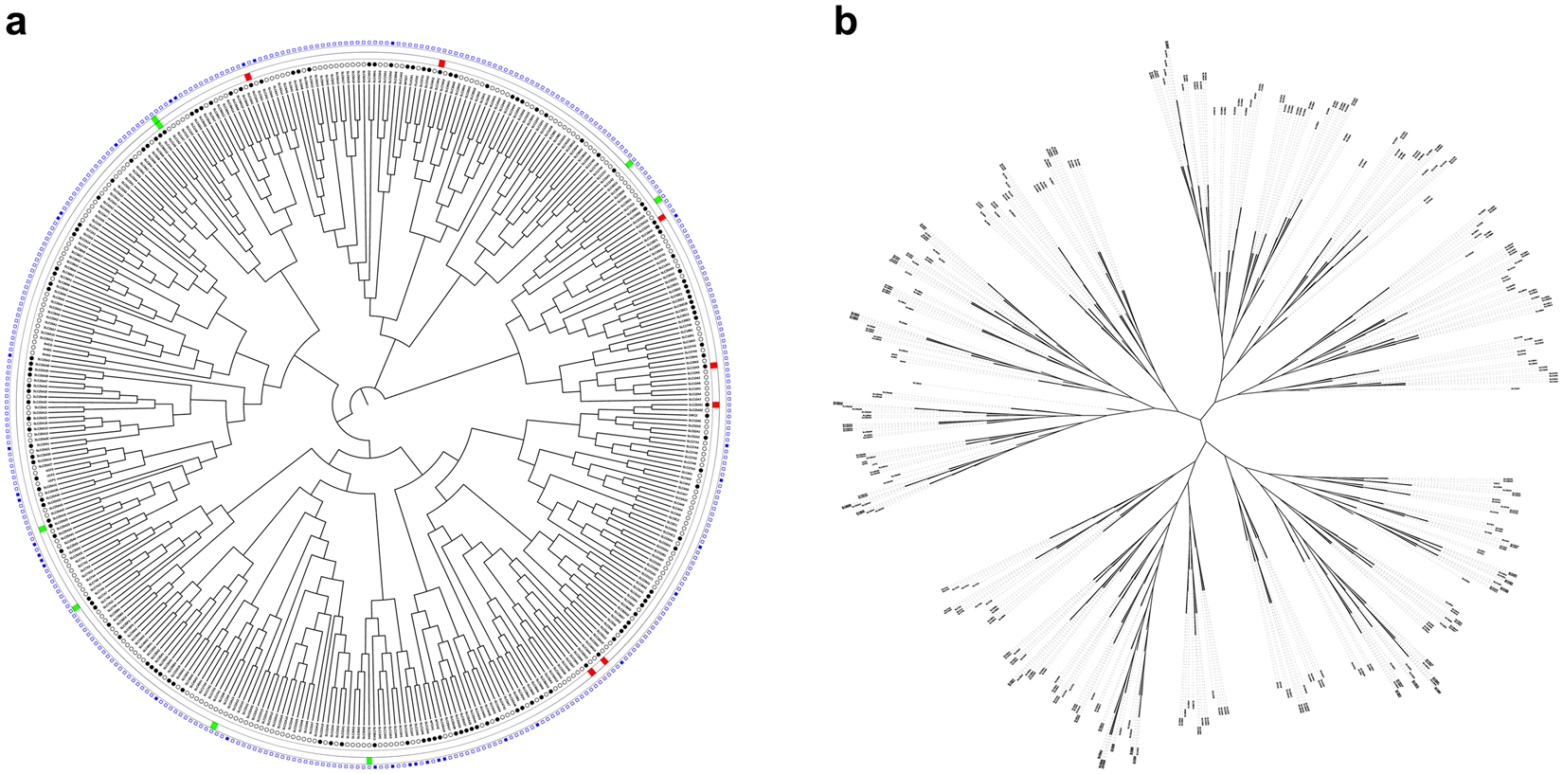
Subsets of SLC transporter proteins are expressed and regulated in primary human neutrophils. Scalable phylogenetic trees in both (**a**) circular and (**b**) unrooted display modes indicate evolutionary relationships between SLC transporter proteins in humans (branch lengths are not proportional to genetic distance; see Supplementary Fig. S1 for trees with proportional branch lengths). In (**a**), for primary human neutrophil expression data (inner circle), expressed proteins are marked with a black dot, whereas proteins not expressed are denoted with a white dot. For GM-CSF (second circle from centre) and TNFα (third circle from centre) regulation data, significant up-regulation of a gene is denoted with a green box, significant down-regulation is denoted by a red box, and a lack of significant change in regulation is unmarked. For human neutrophil proteomics data (outer circle), any proteins identified in one or more of the analyses are denoted with a blue box, and any not identified are denoted with a white box.

To characterise the expression and regulation of transporter proteins in human neutrophils, we interrogated the best publicly-available RNAseq dataset from human neutrophils^21^. Initially we examined the SLC family, which are thought to be responsible for drug influx, and identified all members expressed in neutrophils. These are shown as a phylogenetic tree to allow visualisation of the evolutionary relationships between the members of transporter protein families in humans (Fig. 1, Supplementary Fig. S1). In primary human neutrophils, 134 of the 389 SLC protein-coding family members (34%) were expressed. In the presence of the best characterised neutrophil survival factor, granulocyte macrophage colony-stimulating factor (GM-CSF), nine genes were significantly regulated, all of which were expressed in primary human neutrophils. Of these genes, two of nine (*SLC1A5* and *SLC25A25*) were up-regulated, whilst the remaining seven genes (*SLC10A3*, *SLC15A3*, *SLC15A4*, *SLC16A14*, *SLC16A6*, *SLC19A1* and *SLC25A51*) were down-regulated. In the presence of tumor necrosis factor alpha (TNFα), another key regulator of neutrophil function, the expression of six genes was significantly up-regulated. Of these, three were expressed in unstimulated primary human neutrophils (*SLC1A5*, *SLC35B2* and *SLC7A5*), whilst three were not expressed (*SLC11A2*, *SLC2A6* and *SLC30A4*). No genes were found to be significantly down-regulated. The regulation of just one gene, *SLC1A5*, was significantly affected by GM-CSF and TNFα, being up-regulated by both mediators. Whilst *SLC1A5* (also known as Alanine Serine Cysteine Transporter 2, *ASCT2*) is a neutral amino acid transporter with ubiquitous expression, overexpression of this gene has been discovered in various cancers^22, 23^, and it is currently being explored as a possible drug target for cancer treatment^24^. To extend these studies, we carried out an analysis of the SLC transporter proteins identified in any of eight publicly available human neutrophil proteomics studies^25–32^. In these datasets, a total of 33 SLC transporter proteins were identified, of which we had identified 20 as being expressed based on RNAseq data, highlighting the incomplete nature of current proteomic datasets and the differences between gene and protein expression in neutrophils. In general, our data indicate significant potential for differential sensitivity of activated neutrophils to different drug classes and should be considered as part of anti-inflammatory drug design.

**Figure 2:**
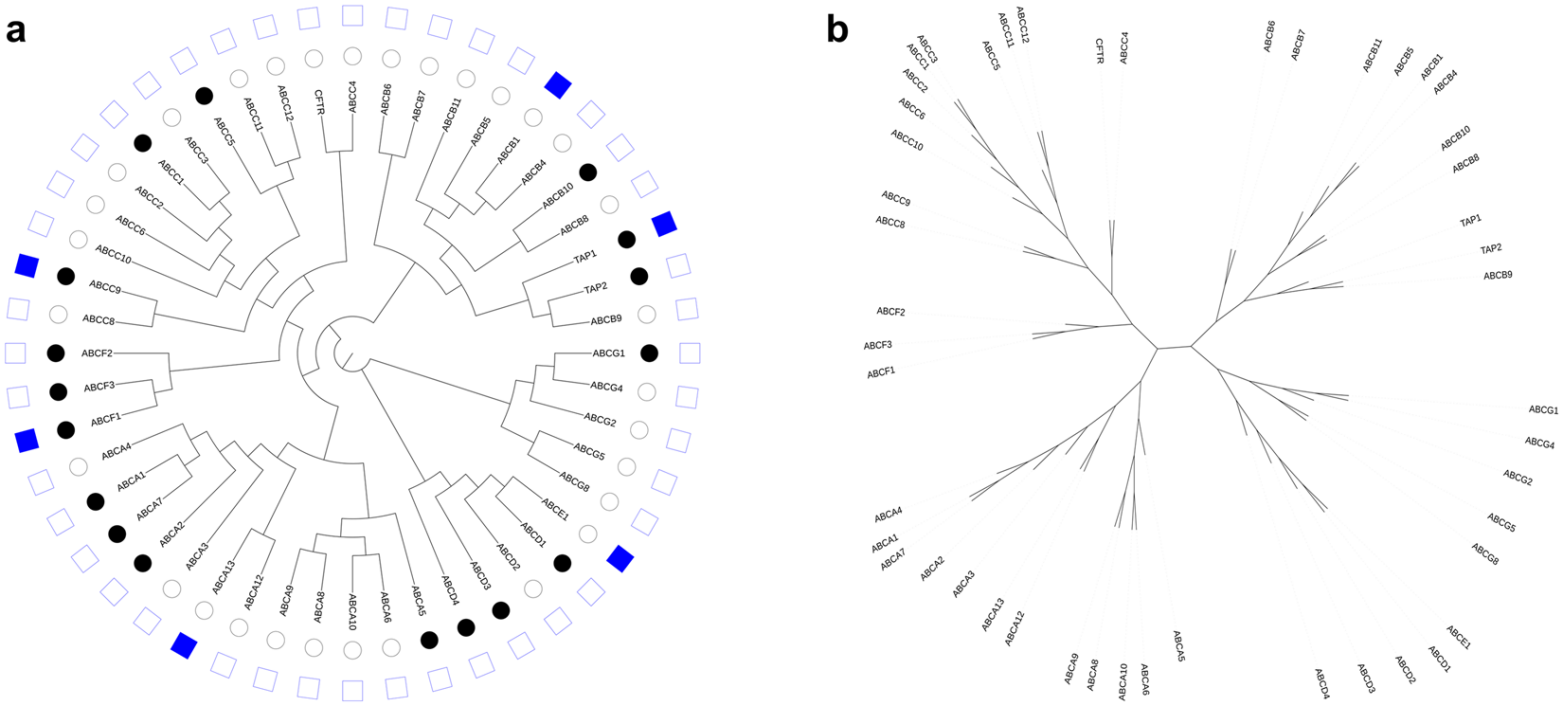
Subsets of ABC transporter proteins are expressed but not regulated in primary human neutrophils. Scalable phylogenetic trees in both (**a**) circular and (**b**) unrooted display modes indicate evolutionary relationships between ABC transporter proteins in humans (branch lengths are not proportional to genetic distance; see Supplementary Fig. S2 for trees with proportional branch lengths). In (**a**), for primary human neutrophil expression data (inner circle), expressed proteins are marked with a black dot, whereas proteins not expressed are denoted with a white dot. For human neutrophil proteomics data (outer circle), any proteins identified in one or more of the analyses are denoted with a blue box, and any not identified are denoted with a white box.

The ABC transporter family is considered to be responsible for drug efflux. A parallel analysis of this family was performed and displayed as a phylogenetic tree in the same way as for the SLC family (Fig. 2, Supplementary Fig. S2). In primary human neutrophils, 17 out of 48 ABC protein-coding family members (35%) were expressed. Interestingly, in contrast to the SLC transporters, none of the ABC transporters were significantly up- or down-regulated in the presence of either GM-CSF or TNFα. This perhaps suggests that the SLC transporters may have a specific role in regulation of neutrophil function in response to these key mediators. Across the various human neutrophil proteomics studies, 6 ABC transporter proteins were identified, 3 of which we identified as being expressed in primary human neutrophils based on RNAseq data.

**Figure 3:**
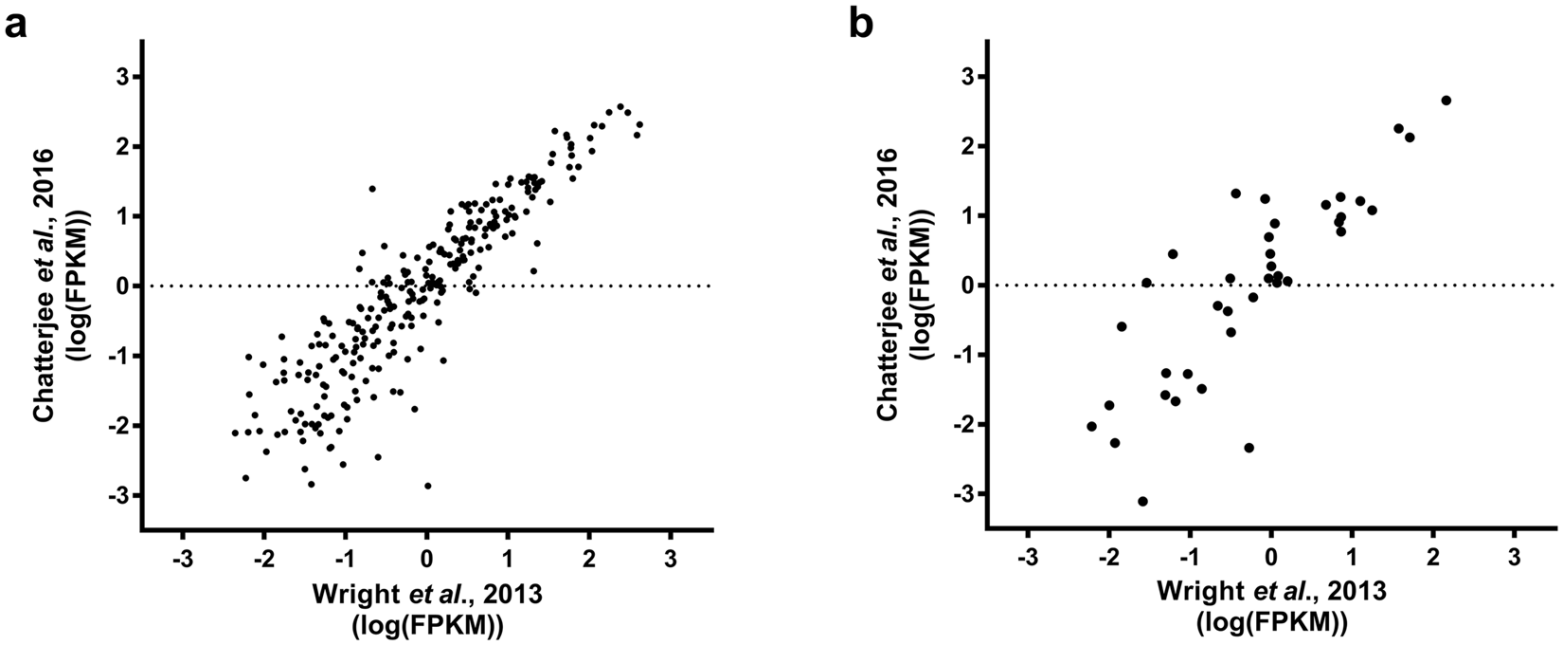
Expression of human SLC and ABC transporter proteins across two different datasets shows highly significant positive correlation. Scattergraphs show comparison of expression of (**a**) SLC and (**b**) ABC transporter proteins in datasets by Wright *et al*. and Chatterjee *et al*.^21, 33^. Data points represent the mean of replicate values. Any genes in the Wright *et al*. dataset which could not be identified in the Chatterjee *et al*. dataset are not shown. For (**a**), r = 0.9157, *P* < 0.0001 (Spearman correlation, n = 368 pairs). For (**b**), r = 0.8368, *P* < 0.0001 (Spearman correlation, n = 47 pairs). Corresponding tables are provided in Supplementary Tables S3 and S4.

In order to validate our approach and the quality of the dataset, we also performed the same unstimulated expression analysis on a second human neutrophil RNAseq dataset^33^. This analysis showed a highly significant positive correlation between the two datasets for both the SLC transporters (r = 0.9157, *P* < 0.0001) and the ABC transporters (r = 0.8368, *P* < 0.0001) (Fig. 3, Supplementary Tables S3 and S4), providing further confidence in our approach.

### Anti-inflammatory compounds differentially accumulate in zebrafish

**Figure 4:**
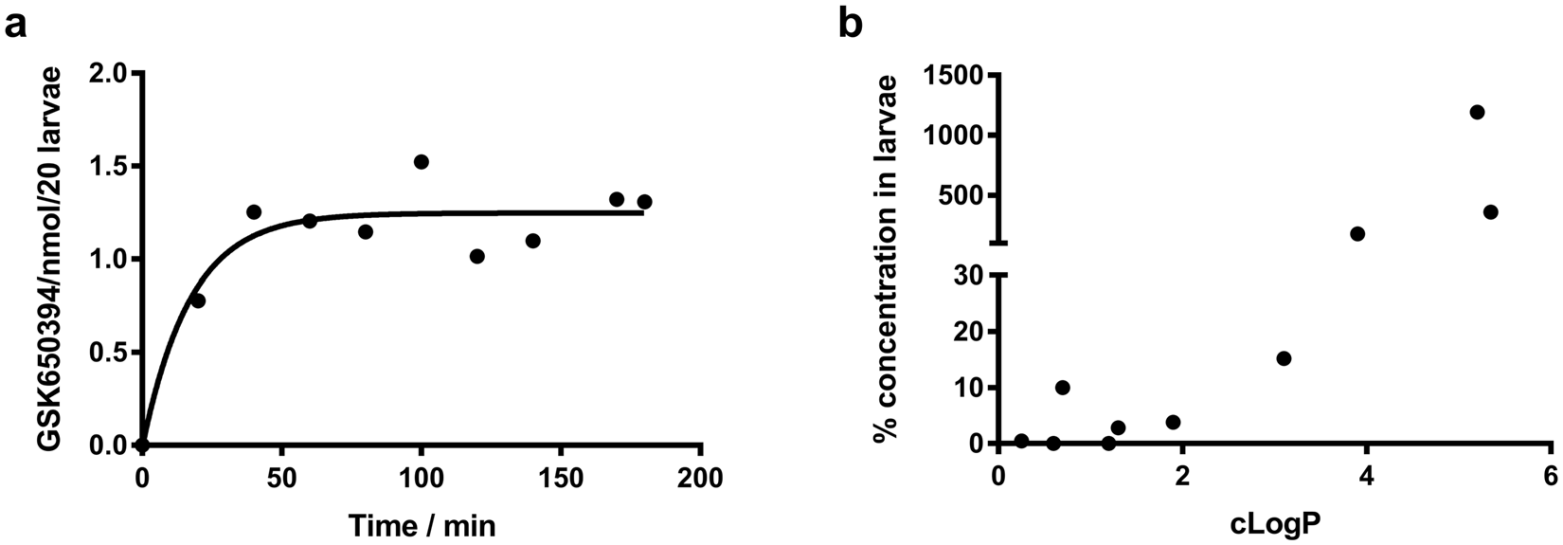
Drug penetration into larvae is not entirely predicted by cLogP. **(a**) Amount of GSK650394 in zebrafish larvae was measured at the timepoints shown by mass spectrometry. (**b**) cLogP data from ref. 36 and the corresponding data for GSK650394 are shown plotted against fold enrichment of the compound in larvae compared to the concentration in surrounding liquid. Data are for larvae at 3 dpf incubated for 1 hour.

Our model of choice for the mechanistic dissection of neutrophil function is the zebrafish^34^. The small size of the larvae combined with the presence of functional innate immunity make this system highly suitable for drug screening for new anti-inflammatory molecules^4, 5, 35^. We have previously shown that the SGK1 inhibitor GSK650394 can induce apoptosis in human neutrophils *in vitro*, and in zebrafish inflammation models *in vivo*. Moreover, mass spectrometry analysis has shown that this compound is able to penetrate zebrafish larvae in order to exert its effect^20^. To examine the kinetics of drug penetration into zebrafish larvae, we performed a timecourse of compound administration and analysed the resulting concentrations by mass spectrometry. Accumulation of GSK650394 in larvae was rapid reaching a plateau within an hour (Fig. 4a). Other groups have examined the ability of compounds to penetrate zebrafish larvae^36, 37^, and reanalysis of published data reveals a marked dichotomy between compounds that are found inside zebrafish larvae at very low concentrations (<15% of external concentration) and compounds which are markedly concentrated within the larvae (>1000%, Fig. 4b). While high cLogP values predicted concentration in larvae, for lower values there was no correlation. This suggested to us that active transport mechanisms might be responsible for some of the differences between rapidly accumulated compounds and compounds excluded from the larvae.

### Zebrafish neutrophils express a distinct subset of transporter proteins

To examine whether drug transporter expression and regulation might underlie the unpredictable nature of larval drug penetration, and to specifically relate this to the neutrophil, we used RNAseq datasets of neutrophils sorted from *Tg*(*mpx:GFP*)*i114* zebrafish at 5 days post fertilisation (dpf)^38^. From these datasets, we examined expression of transporter gene families. Zebrafish orthologues were identified by manually searching the Ensembl database for the orthologue of each human SLC and ABC gene. Furthermore, to ensure that all relevant zebrafish genes were included, the paralogous genes for each zebrafish orthologue were identified using Ensembl. These were then cross-referenced against our existing dataset, and any additional genes not yet in our dataset were added. Where it was unclear if a particular paralogue was a ‘true’ paralogue, the corresponding gene tree in Ensembl was examined. If a paralogue had a clear relationship to one or more annotated drug transporter genes in zebrafish, it was included in our dataset. Any paralogues which could not be identified as having such a relationship, or which were clearly related to different protein families, were excluded from our analysis.

**Figure 5:**
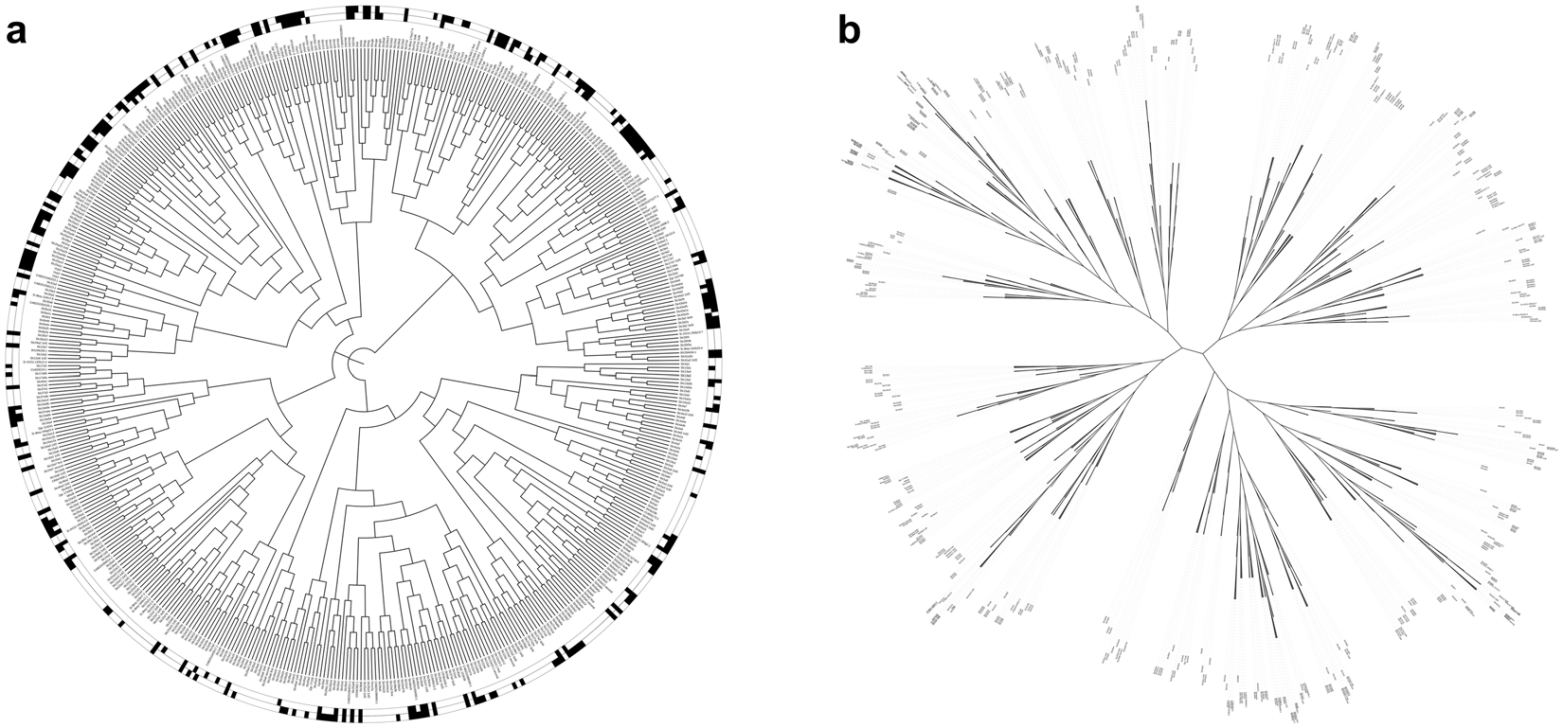
Zebrafish neutrophils and non-neutrophil cells express distinct subsets of SLC transporter proteins. Scalable phylogenetic trees in both (**a**) circular and (**b**) unrooted display modes indicate evolutionary relationships between SLC transporter proteins in zebrafish (branch lengths are not proportional to genetic distance; see Supplementary Fig. S5 for trees with proportional branch lengths). In (**a**), for both neutrophil (inner circle) and background cell (outer circle) expression data, expressed proteins are marked with a black box, whereas proteins not expressed are unmarked.

For the zebrafish SLC genes and their paralogues, a phylogenetic tree was constructed to display their ancestral relationships (Fig. 5, Supplementary Fig. 5). Of the 533 zebrafish protein-coding genes, 142 (27%) were expressed in zebrafish larvae neutrophils, whilst 219 (41%) were expressed in “background” (non-neutrophil) cells. 12 of the 142 genes expressed in neutrophils were expressed solely in neutrophils, and not in background cells: *slc2a3b*, *slc2a8*, *slc2a12*, *slc2a15a*, *slc3a2_4of4*, *slc4a2b*, *slc13a1*, *slc13a5b*, *slc34a2b*, *slco3a1*, *rhcgl1*, and *CR352249.1*. A further 130 genes were expressed in both neutrophil and background cells, whilst 89 genes were expressed in background cells yet not in neutrophils at this developmental stage.

**Figure 6:**
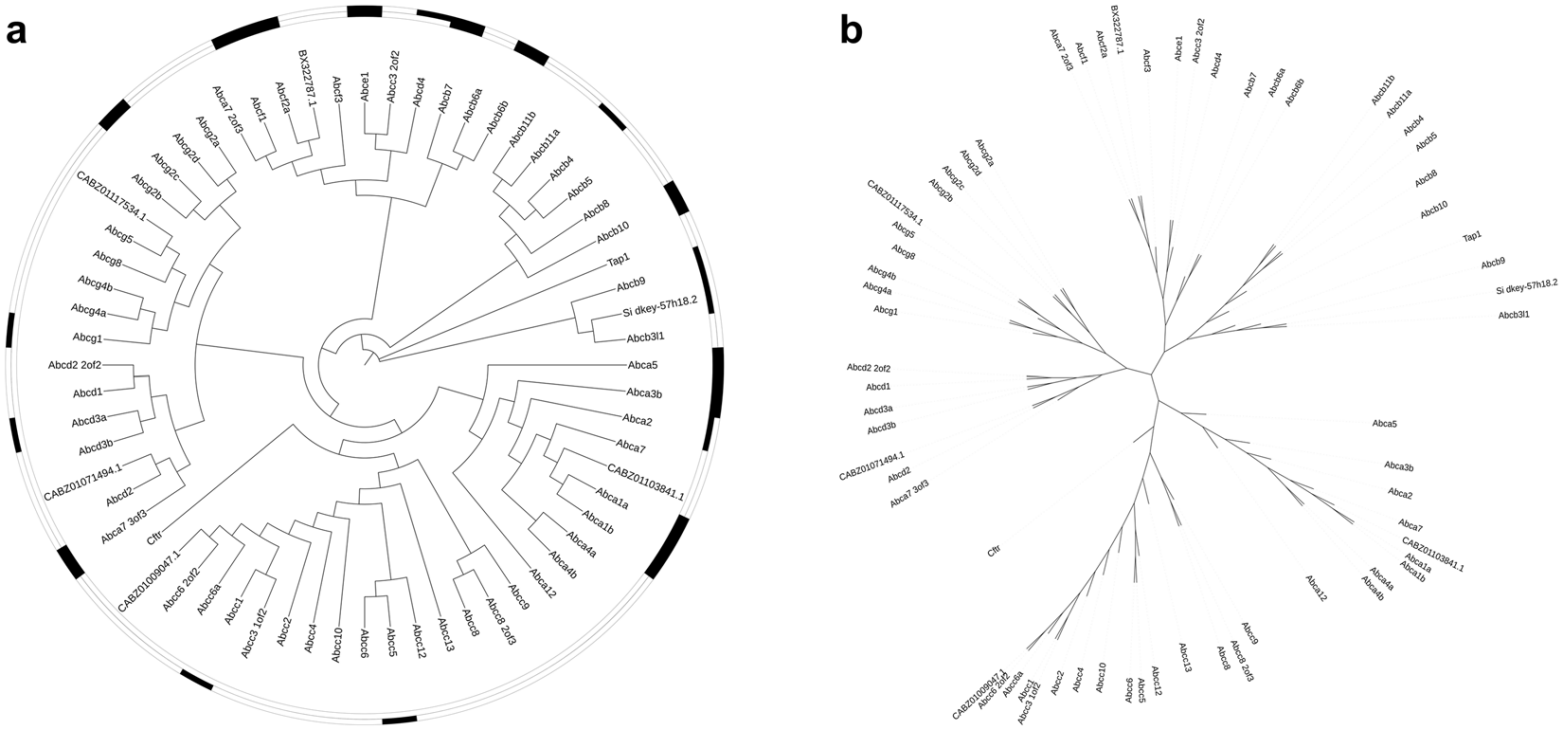
Zebrafish neutrophils and non-neutrophil cells express distinct subsets of ABC transporter proteins. Scalable phylogenetic trees in both (**a**) circular and (**b**) unrooted display modes indicate evolutionary relationships between ABC transporter proteins in zebrafish (branch lengths are not proportional to genetic distance; see Supplementary Fig. S6 for trees with proportional branch lengths). In (**a**), for both neutrophil (inner circle) and background cell (outer circle) expression data, expressed proteins are marked with a black box, whereas proteins not expressed are unmarked.

Finally, a phylogenetic tree was produced to examine the ABC transporter family in zebrafish (Fig. 6, Supplementary Fig. 6). There are 64 protein-coding ABC transporter genes in total, of which 16 genes (25%) were found to be expressed in zebrafish neutrophils, and 17 genes (27%) were expressed in background cells. Of the 16 genes expressed in neutrophils, four were expressed in neutrophils alone: *abca2*, *abcb4*, *abcb9*, and *si:dkey-57h18.2*. In addition, 12 genes were expressed in both neutrophil and background cells, whilst five genes were expressed in non-neutrophils cells only.

### Defined SLC and ABC drug transporters are conserved in human and zebrafish neutrophils

A more detailed analysis of available datasets revealed that the overall proportion of drug transporter proteins expressed in neutrophils of each species was approximately equal (considering the various technical differences between the datasets). For the SLC family, 34% of human SLC genes were expressed in human neutrophils, compared with 27% of zebrafish SLC genes expressed in zebrafish neutrophils. Similarly, for the ABC family, 35% of ABC genes in humans were expressed in neutrophils, compared to 25% expression in zebrafish neutrophils. In addition, in both human and zebrafish neutrophils, the median SLC and ABC expression values were roughly in line with the median expression level of all genes in the human and neutrophil datasets respectively. In humans, the median ‘fragments per kilobase of exon per million fragments mapped’ (FPKM) values for SLC and ABC genes were 0.22 and 0.34, compared with a median FPKM value of 0.26 for the entire set of genes. Likewise, in zebrafish neutrophils, the median FPKM values were 0.16 for SLC genes, 0.13 for ABC genes, and 0.10 for the entire gene set. Furthermore, we found that for the SLC drug transporters, around half of the genes expressed in primary human neutrophils (65 out of 134 genes, 49%) had at least one orthologous gene in zebrafish which was also expressed in zebrafish neutrophils (see Supplementary Table S7 for a full list of genes). In the set of ABC drug transporters, a similar proportion of the genes expressed in human neutrophils (eight out of 17 genes, 47%) had an orthologous gene also expressed in zebrafish neutrophils (full list provided in Supplementary Table S8). When taken together, all these data suggest a level of conservation in SLC and ABC drug transporter expression between human and zebrafish neutrophils.

## Discussion

The drug discovery process optimises the compound properties such that compounds can reach their target, act on it to have the desired therapeutic effect, and be metabolised and excreted in such a way as to avoid toxicity. This is crucial both for discovery science in animal models and for compounds that ultimately make it to the clinic. Large compound screens in zebrafish, whether of approved drugs, natural products, new chemical entities or tool compounds, have been highly successful (for a review of zebrafish drug screens see MacRae and Peterson^39^). However, a proportion of potentially valuable compounds or drug targets may be missed due to the “false negative” rate of these approaches^40^.

“False negatives” may occur due to the inherent biological variation of the experimental approach, solubility issues or the fact that the compound cannot reach its intracellular target. In the latter case, we suggest that differential drug transporter expression may hold the key to the failure of these compounds. In cases where drugs are toxic, one possible cause is that the drug (or its metabolites) is not actively removed from the cell. Drug transporters (particularly ABC transporters) appear to be crucial in eliminating this source of toxicity, as shown by a reduced tolerance of patients with drug transporter mutations to some medications^14, 15^.

Drug transporters originate from two gene families, the SLC and ABC families, and are responsible for the transport of metabolites and ions. Drugs which are structurally similar to endogenous metabolites can hijack the corresponding transporters to gain entry to or exit from a cell. This idea has been described and evidence presented in the literature for around 20 years^7, 41, 42^, yet the importance of these drug transporters in the drug discovery process is often neglected. This is largely due to the substantial number of transporters in the genome, the fact that in most cases multiple transporters are each able to transport several drugs^8^, and the limited knowledge of which transporters are utilised by a particular drug.

We propose that a more thorough understanding of drug transporter expression in the cell type, organ or animal of interest may go some way towards addressing the issue of compound entry into and exit from a cell, making drug screening an even more efficient approach to drug discovery. We have begun this process, by undertaking a comprehensive analysis of drug transporter expression in the neutrophil. We have also explored expression in a model organism for immune-based *in vivo* compound screens, the zebrafish.

Using the human gene families of SLC and ABC transporters as a starting point, we probed a published dataset to assess transporter expression in primary human neutrophils^21^. This revealed expression of 134 (of 389) SLC transporters and 17 (of 48) ABC transporters in human neutrophils. Of these, two and three SLC transporters were up-regulated by GM-CSF and TNFα, respectively. One transporter, *SLC1A5*, was up-regulated by both stimuli. ABC transporter expression was not regulated by these stimuli in this dataset. Moreover, when we compared these expression data to those in a second dataset^33^, we found that the relative expression patterns were largely conserved, thereby validating our results.

In the zebrafish, we searched for the orthologues of the human gene family members and further analysed the paralogues yet to be assigned to the family by manual analysis of the gene trees present in Ensembl. This uncovered a number of previously unannotated paralogues in both the zebrafish SLC and ABC transporter families. Probing of the RNAseq dataset from sorted zebrafish larval neutrophils (GFP positive) and background cells (GFP negative), revealed expression of 231 SLC transporters in zebrafish larvae, including 12 SLC transporters specifically in neutrophils. Of the smaller ABC family, 21 transporters were expressed in zebrafish larvae and four of these specifically in neutrophils. In addition, a general comparison of the human and zebrafish larvae expression data using our chosen cut-off values indicated that the proportion of SLC and ABC transporter genes expressed in neutrophils was broadly conserved between species. However, it should be considered that these data correspond to zebrafish larvae at an early stage of development (5 dpf), and that such expression is likely to change as the zebrafish mature.

In neutrophils, mRNA expression and protein expression may not always correlate. Neutrophils must act rapidly to kill infectious threats and their arsenal must be preformed. Thus, many highly abundant proteins might not be expressed at mRNA level in mature neutrophils^31^. While the two human neutrophil RNAseq datasets that we have probed are largely consistent, a summary by Tomazella *et al*.^25^ demonstrated that human neutrophil proteomic datasets are highly variable among samples^25^. In addition, in both the human and zebrafish datasets, we have studied resting, rather than inflammatory, neutrophils. In the human dataset, some transporters show regulation in the presence of an inflammatory stimulus, suggesting that transporter expression may be modified in inflammatory states. However, extracting neutrophils from inflammatory sites is technically complex, particularly in zebrafish. Such neutrophil asynchrony and plasticity, including their ability to release granule contents at the cell surface promptly by exocytosis, endocytosis, proteolysis and ectosome formation, are important considerations here, and have been reviewed extensively elsewhere^43–46^.

Here, we present RNA expression data alongside phylogenetic analysis which we hope will be easy to navigate and prove to be an important resource for the field. The key to now using this tool to improve the drug discovery process is to understand which metabolites use which SLC and ABC transporters, and in turn to use this knowledge to inform compound design to more closely mimic metabolites, where possible. *In silico* comparison of drugs and their metabolite-likenesses would help to form hypotheses of potential transporters which could be tested in the zebrafish model. One possible experimental approach would be to knock out a family of SLC transporters using CRISPR/Cas9 genome editing, and then assess the effect of this on drug penetration into neutrophils or zebrafish larvae using a well-defined phenotypic readout. There is not currently a model system for rational dissection of drug transporters and their effects on inflammation, but we anticipate that this resource will provide the first step towards building such a system *in vivo*.

These are not small tasks, but will be hugely beneficial to those using zebrafish and neutrophils for drug discovery. We believe the genetic tractability, ready accessibility to externally-added compounds, and range of transgenic and biological assays of the zebrafish provide an ideal model for the molecular dissection of drug transporter biology *in vivo*.

## Methods

### Phylogenetic analysis

Lists of the SLC and ABC transporter genes found in humans were obtained from the HGNC database (April 2015)^9^. The Ensembl database (human data: version GRCh38.p2, release 79; zebrafish data: version Zv9, release 79)^47^ was searched manually for each human SLC and ABC gene identifier, and orthologous genes present in the zebrafish (*Danio rerio*) database (version Zv9, release 79) were identified. Due to some observed discrepancies in the naming of the zebrafish orthologues (for example, where two different genes had identical names in the database), the unique numerical identifier (for example, *ENSDARG00000000001*) was used to search for orthologues in each case, to avoid ambiguity. Gene names as returned from Ensembl were generally retained for all further analysis. Genes with identical names were arbitrarily given suffixes of *_first* and *_second* to enable distinction. Where genes were identified as being a ‘novel gene’ in the Ensembl database with no additional annotation, the corresponding name for the same gene was obtained from an earlier release of the same version of Ensembl (version Zv9, release 75) in accordance with the RNAseq data used (under accession number GSE78954).

In both human and zebrafish datasets, for each individual gene the longest corresponding protein-coding sequence was obtained from the Ensembl database. Any genes which were found not to code for proteins were then excluded from the analysis. Regulatory genes and pseudogenes were also excluded. Files containing protein sequence data used in this work can be accessed at The University of Sheffield Research Data Catalogue and Repository, ORDA (https://sheffield.figshare.com).

Clustal Omega (version 1.2.3) was used for multiple sequence alignment of protein sequences^48, 49^. The ‘Phylogenetic Tree’ output (.ph file) was converted to Newick format using the Phylogeny.fr resource^50^. The latter format was inputted into the Interactive Tree of Life (iTOL) programme (version 3.3) to produce phylogenetic trees displaying the evolutionary relationships in the human and zebrafish SLC and ABC families^51^. The phylogenetic trees were then annotated with the corresponding expression and proteomics data.

### Expression and regulation of human transporter proteins

Published transcriptomics datasets (accessed *via* the NCBI Gene Expression Omnibus (GEO), accession number GSE40548^21^) were downloaded and used for analysis. In these data, RNA from 3 different neutrophil donors was analysed, isolated from 3 × 10^7^ neutrophils, and in all cases RNA integrity was ≥8.0. A gene was herein defined as being expressed if it had a FPKM value greater than or equal to 1, a conservative value chosen due to its relationship to expression of myeloperoxidase (MPO) - a well-validated neutrophil protein (expression level 0.98 FKPM). Any gene with a FPKM value less than 1 was defined as unexpressed. Human neutrophil proteomics data from 8 different studies were manually searched for any SLC and ABC transporter proteins identified therein^25–32^. For expression validation, each SLC and ABC gene was searched for in the dataset generated by Chatterjee *et al*. (4 donors, median RNA integrity 8.05) (accession number GSE59528)^33^, and mean FPKM values were plotted against the corresponding data from Wright *et al*. (following logarithmic transformation of both datasets)^21^. All human genes and proteins are referred to according to HGNC conventions throughout. Raw data files used to generate figures in this work can be accessed at ORDA.

### Expression and regulation of zebrafish transporter proteins

Cells from zebrafish larvae (*Tg(mpx:GFPi114)*, 5 dpf) were sorted into GFP-positive (neutrophils) and GFP-negative (background) cell populations and RNAseq analysis performed, as described previously^38^ (data deposited on GEO under accession number GSE78954). Each experiment was performed in duplicate. For both neutrophil and background cell expression levels, we define any gene with a FPKM value greater than or equal to 1 as being expressed, and any with a FPKM value less than 1 as being unexpressed, in line with the definitions used for expression of human genes. All zebrafish genes and proteins are hereby referred to in accordance with the Zebrafish Model Organism Database (ZFIN) naming conventions as far as practicably possible. Raw data files used to generate figures in this work can be accessed at ORDA.

### Mass Spectrometric analysis

Frozen 3 dpf zebrafish samples (20 fish per time point) were defrosted at room temperature on the day of analysis and diluted by the addition of 500 µL water. Each sample was then homogenised using a Soniprep 150 to disrupt the tissue with 3 × 10 s sonication bursts. Samples were subsequently processed then analysed by LC-MS/MS using an API 4000 triple quadrupole mass spectrometer (AB Sciex), as previously described^20^.

## Electronic supplementary material

Supplementary Information

## References

[1] Serhan CN (2007). Resolution of inflammation: state of the art, definitions and terms. FASEB J..

[2] Henry KM, Loynes CA, Whyte MK, Renshaw SA (2013). Zebrafish as a model for the study of neutrophil biology. J. Leukoc. Biol..

[3] d’Alençon CA (2010). A high-throughput chemically induced inflammation assay in zebrafish. BMC Biol..

[4] Robertson AL (2014). A zebrafish compound screen reveals modulation of neutrophil reverse migration as an anti-inflammatory mechanism. Sci. Transl. Med..

[5] Wang X (2014). Inhibitors of neutrophil recruitment identified using transgenic zebrafish to screen a natural product library. Dis. Model. Mech..

[6] Robertson AL (2016). Identification of benzopyrone as a common structural feature in compounds with anti-inflammatory activity in a zebrafish phenotypic screen. Dis. Model. Mech..

[7] Al-Awqati Q (1999). One hundred years of membrane permeability: does Overton still rule?. Nat. Cell Biol..

[8] Dobson PD, Kell DB (2008). Carrier-mediated cellular uptake of pharmaceutical drugs: an exception or the rule?. Nat. Rev. Drug Discov..

[9] Gray KA, Yates B, Seal RL, Wright MW, Bruford EA (2015). Genenames.org: the HGNC resources in 2015. Nucleic Acids Res..

[10] Sahoo S, Aurich MK, Jonsson JJ, Thiele I (2014). Membrane transporters in a human genome-scale metabolic knowledgebase and their implications for disease. Front. Physiol..

[11] O’Hagan S, Kell DB (2015). Understanding the foundations of the structural similarities between marketed drugs and endogenous human metabolites. Front. Pharmacol..

[12] Spratlin J (2004). The absence of human equilibrative nucleoside transporter 1 is associated with reduced survival in patients with gemcitabine-treated pancreas adenocarcinoma. Clin. Cancer Res..

[13] Kobayashi H (2012). Human equilibrative nucleoside transporter 1 expression predicts survival of advanced cholangiocarcinoma patients treated with gemcitabine-based adjuvant chemotherapy after surgical resection. Ann. Surg..

[14] Cascorbi I (2006). Role of pharmacogenetics of ATP-binding cassette transporters in the pharmacokinetics of drugs. Pharmacol. Ther..

[15] Noguchi K, Katayama K, Sugimoto Y (2014). Human ABC transporter ABCG2/BCRP expression in chemoresistance: basic and clinical perspectives for molecular cancer therapeutics. Pharmgenomics Pers. Med..

[16] Inui KI, Masuda S, Saito H (2000). Cellular and molecular aspects of drug transport in the kidney. Kidney. Int..

[17] Dean M, Rzhetsky A, Allikmets R (2001). The human ATP-binding cassette (ABC) transporter superfamily. Genome Res..

[18] Loynes CA (2010). Pivotal Advance: Pharmacological manipulation of inflammation resolution during spontaneously resolving tissue neutrophilia in the zebrafish. J. Leukoc. Biol..

[19] Wardle DJ (2011). Effective caspase inhibition blocks neutrophil apoptosis and reveals Mcl-1 as both a regulator and a target of neutrophil caspase activation. PLoS One.

[20] Burgon J (2014). Serum and glucocorticoid–regulated kinase 1 regulates neutrophil clearance during inflammation resolution. J. Immunol..

[21] Wright HL, Thomas HB, Moots RJ, Edwards SW (2013). RNA-seq reveals activation of both common and cytokine-specific pathways following neutrophil priming. PLoS One.

[22] Fuchs BC, Bode BP (2005). Amino acid transporters ASCT2 and LAT1 in cancer: Partners in crime?. Sem. Cancer Biol..

[23] Liu Y (2015). High expression of Solute Carrier Family 1, member 5 (SLC1A5) is associated with poor prognosis in clear-cell renal cell carcinoma. Sci. Rep..

[24] Bhutia YD, Babu E, Ramachandran S, Ganapathy V (2015). Amino acid transporters in cancer and their relevance to “glutamine addiction”: novel targets for the design of a new class of anticancer drugs. Cancer Res..

[25] Tomazella GG (2009). Proteomic analysis of total cellular proteins of human neutrophils. Proteome Sci..

[26] Lominadze G (2005). Proteomic analysis of human neutrophil granules. Mol. Cell. Proteomics.

[27] Castro MD (2006). Proteome analysis of resting human neutrophils. Protein Peptide Lett.

[28] Jethwaney D (2007). Proteomic analysis of plasma membrane and secretory vesicles from human neutrophils. Proteome Sci..

[29] Uriarte SM (2008). Comparison of proteins expressed on secretory vesicle membranes and plasma membranes of human neutrophils. J. Immunol..

[30] Xu P (2009). Subproteome analysis of the neutrophil cytoskeleton. Proteomics.

[31] Rørvig S, Østergaard O, Heegaard NHH, Borregaard N (2013). Proteome profiling of human neutrophil granule subsets, secretory vesicles, and cell membrane: correlation with transcriptome profiling of neutrophil precursors. J. Leukoc. Biol..

[32] Zhou J-Y (2013). Trauma-associated human neutrophil alterations revealed by comparative proteomics profiling. Proteomics Clin. Appl..

[33] Chatterjee A, Stockwell PA, Rodger EJ, Morison IM (2016). Genome-scale DNA methylome and transcriptome profiling of human neutrophils. Sci. Data.

[34] Renshaw SA (2006). A transgenic zebrafish model of neutrophilic inflammation. Blood.

[35] Hall CJ (2014). Repositioning drugs for inflammatory disease – fishing for new anti-inflammatory agents. Dis. Model. Mech.

[36] Berghmans S (2008). Zebrafish based assays for the assessment of cardiac, visual and gut function — potential safety screens for early drug discovery. J. Pharmacol. Toxicol. Methods.

[37] Ordas A (2015). Testing tuberculosis drug efficacy in a zebrafish high-throughput translational medicine screen. Antimicrob. Agents. Chemother..

[38] Rougeot, J. *et al*. In *Host-Bacteria Interactions:* Methods *and Protocols* (eds Annette C. Vergunst, A. C. & O’Callaghan, D.) 261–274 (Springer New York, 2014).

[39] MacRae CA, Peterson RT (2015). Zebrafish as tools for drug discovery. Nat. Rev. Drug Discov..

[40] Malo N, Hanley JA, Cerquozzi S, Pelletier J, Nadon R (2006). Statistical practice in high-throughput screening data analysis. Nat. Biotechnol..

[41] Sai Y, Tsuji A (2004). Transporter-mediated drug delivery: recent progress and experimental approaches. Drug Discov.Today.

[42] Dobson PD, Lanthaler K, Oliver SG, Kell DB (2009). Implications of the dominant role of transporters in drug uptake by cells. Curr. Top. Med. Chem..

[43] Futosi K, Fodor S, Mócsai A (2013). Neutrophil cell surface receptors and their intracellular signal transduction pathways. Int. Immunopharmacol..

[44] Nauseef WM, Borregaard N (2014). Neutrophils at work. Nat. Immunol..

[45] Margaroli C, Tirouvanziam R (2016). Neutrophil plasticity enables the development of pathological microenvironments: implications for cystic fibrosis airway disease. Mol. Cell. Pediatr..

[46] Wiedow O, Meyer-Hoffert U (2005). Neutrophil serine proteases: potential key regulators of cell signalling during inflammation. J. Intern. Med..

[47] Cunningham F (2015). Ensembl 2015. Nucleic Acids Res..

[48] Sievers F (2011). Fast, scalable generation of high‐quality protein multiple sequence alignments using Clustal Omega. Mol. Syst. Biol..

[49] Goujon M (2010). A new bioinformatics analysis tools framework at EMBL–EBI. Nucleic Acids Res..

[50] Dereeper A (2008). Phylogeny.fr: robust phylogenetic analysis for the non-specialist. Nucleic Acids Res..

[51] Letunic I, Bork P (2016). Interactive tree of life (iTOL) v3: an online tool for the display and annotation of phylogenetic and other trees. Nucleic Acids Res..

