# Supplementary material for: Expression and regulation of drug transporters in vertebrate neutrophils
Source: Sci Rep. 2017 Jul 10;7:4967. doi: 10.1038/s41598-017-04785-4 (PMC5504015; doi:10.1038/s41598-017-04785-4)
Supplement: Supplementary file 1 — Supplementary Information [file 41598_2017_4785_MOESM1_ESM.pdf]

Supplementary Information (SI)

Expression and regulation of drug transporters in vertebrate neutrophils

Matthew J. Foulkes[1,2,3,*], Katherine M. Henry[1,3,*], Julien Rougeot[4], Ed Hooper-Greenhill[5], Catherine A. Loynes[1, 3], Phil Jeffrey[6], Angeleen Fleming[7,8], Caroline O. Savage[5], Annemarie H. Meijer[4], Simon Jones[2] & Stephen A. Renshaw[1,3]

[1] The Bateson Centre, The University of Sheffield, Sheffield, S10 2TN, UK.
[2] Department of Chemistry, The University of Sheffield, Sheffield, S3 7HF, UK.
[3] Department of Infection, Immunity & Cardiovascular Disease, The University of Sheffield, Sheffield, S10 2RX, UK.
[4] Institute of Biology, University of Leiden, 2333 CC, Leiden, The Netherlands.
[5] Immuno-Inflammation Therapy Area Unit, GlaxoSmithKline Research and Development Ltd., Stevenage, SG1 2NY, UK.
[6] Rare Diseases Research Unit, Pfizer Ltd., Cambridge, CB21 6GP, UK.
[7] Department of Physiology, Development and Neuroscience, University of Cambridge, Cambridge, CB2 3EG, UK.
[8] Department of Medical Genetics, University of Cambridge, Cambridge Institute for Medical Research, Addenbrooke's Hospital, Cambridge, CB2 2XY, UK.

*These authors contributed equally to this work.
Correspondence and requests for materials should be addressed to S.A.R. (email: s.a.renshaw@sheffield.ac.uk).

Supplementary Figure S1. Subsets of SLC transporter proteins are expressed and regulated in primary human neutrophils.

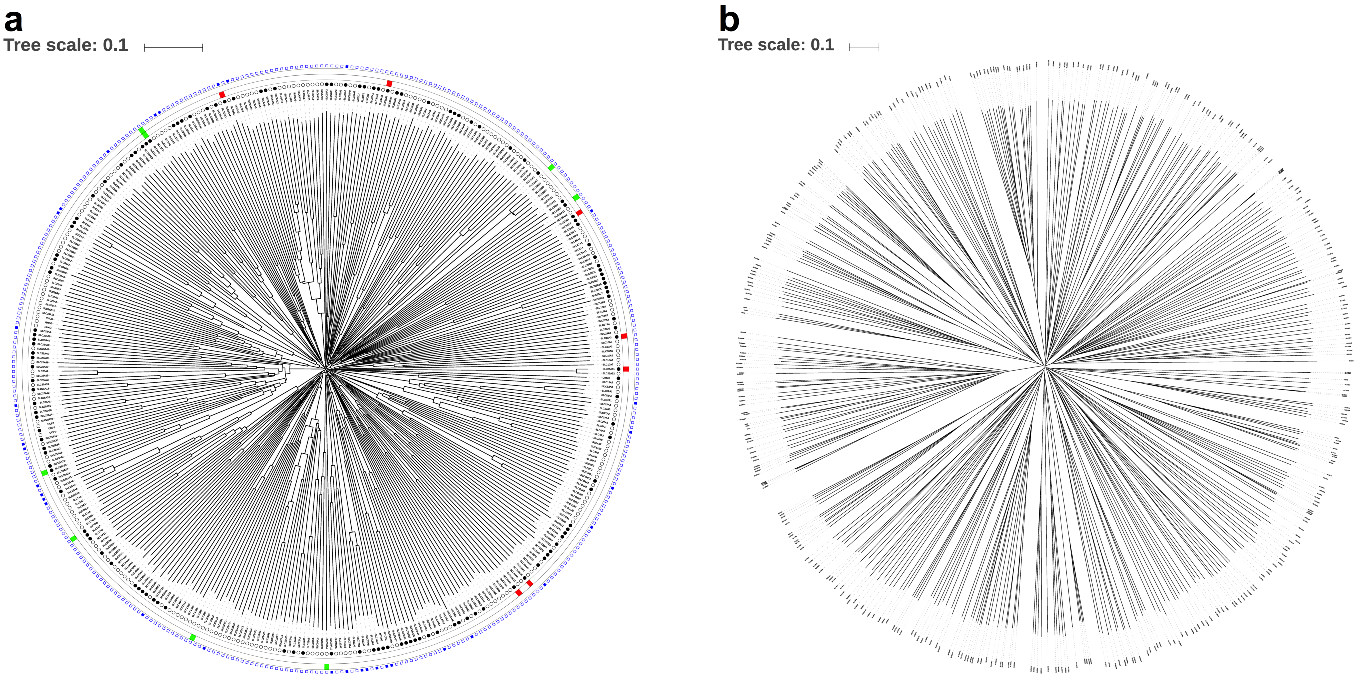

Scalable phylogenetic trees in both (**a**) circular and (**b**) unrooted display modes indicate evolutionary relationships between SLC transporter proteins in humans. Branch lengths are proportional to genetic distance. In (**a**), for primary human neutrophil expression data (inner circle), expressed proteins are marked with a black dot, whereas proteins not expressed are denoted with a white dot. For GM-CSF (second circle from centre) and TNFα (third circle from centre) regulation data, significant up-regulation of a gene is denoted with a green box, significant down-regulation is denoted by a red box, and a lack of significant change in regulation is unmarked. For human neutrophil proteomics data (outer circle), any proteins identified in one or more of the analyses are denoted with a blue box, and any not identified are denoted with a white box.

Supplementary Figure S2. Subsets of ABC transporter proteins are expressed but not regulated in primary human neutrophils.

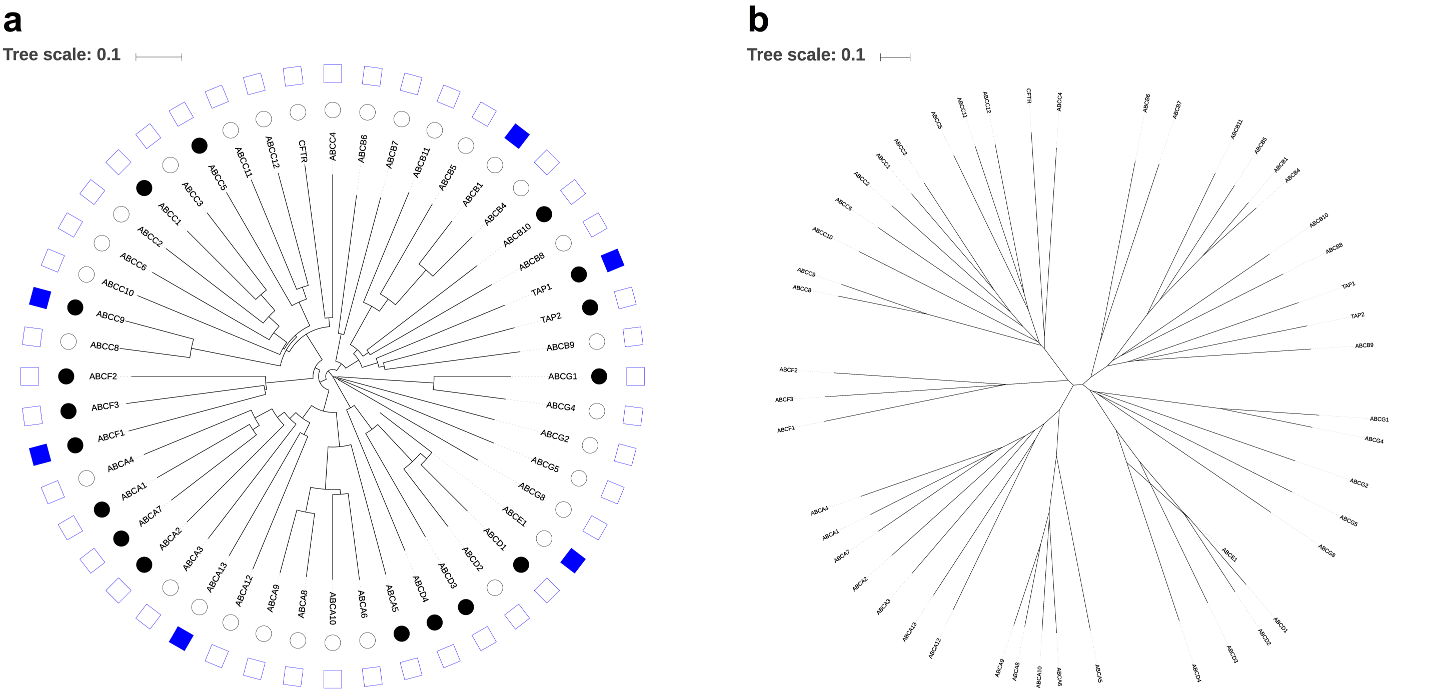

Scalable phylogenetic trees in both (**a**) circular and (**b**) unrooted display modes indicate evolutionary relationships between ABC transporter proteins in humans. Branch lengths are proportional to genetic distance. In (**a**), for primary human neutrophil expression data (inner circle), expressed proteins are marked with a black dot, whereas proteins not expressed are denoted with a white dot. For human neutrophil proteomics data (outer circle), any proteins identified in one or more of the analyses are denoted with a blue box, and any not identified are denoted with a white box.

Supplementary Table S3. Expression of SLC transporter proteins is largely consistent across two different datasets.

SLC transporter	Expression (FPKM)	
	Wright *et al.*, 2013	Chatterjee *et al.*, 2016
SLC16A3	416.071	206.7938
SLC2A3	384.863	146.301
SLC25A37	365.439	**No data**
SLC44A2	296.96	305.502
SLC11A1	241.006	375.1498
SLC6A6	174.964	308.898
SLC43A2	142.397	195.5783
SLC15A3	114.121	203.8168
SLC12A6	107.268	86.26105
SLC15A4	101.968	132.1664
SLC38A2	73.4412	51.29513
SLC45A4	62.3642	34.80962
SLC25A3	60.4501	74.8113
SLC31A2	59.4817	107.9547
MTCH1	58.9362	96.06843
SLC19A1	56.8214	50.80168
SLCO3A1	53.1765	134.5328
SLC12A9	52.23	147.5515
SLC40A1	37.6849	166.2416
UCP2	35.2824	77.73278
SLC25A44	33.7158	58.39863
SLC20A1	33.0773	16.14776
SLC9A1	25.9958	31.72053
SLC22A4	24.431	30.64313
SLC25A28	23.1132	26.53685
DIRC2	22.9076	4.079744
SLC39A1	21.6872	24.02363
SLC22A18	21.182	36.2852
SLC38A10	21.1819	30.27238
SLC7A5	20.6468	1.637648
SLC9A8	19.8604	18.75233
SLC25A5	19.6541	35.83148
SLC29A1	17.9369	36.96945
SLC3A2	17.5905	22.47888
SLC25A11	17.5471	25.77163

SLC8B1	16.841	11.64638
SLC7A7	16.7696	31.3496
SLC16A5	14.6446	30.8605
SLC35A2	12.2554	9.707798
SLC8A1	12.0016	10.08038
SLC16A6	11.2322	5.730955
SLC35A4	11.1066	13.25903
SLC39A7	10.6539	34.74275
SLC25A51	10.198	10.5348
SLC35C2	10.005	28.4959
SLC35E1	9.68586	**No data**
SLC36A1	9.58739	8.930435
SLC10A3	9.23775	11.80692
SLC36A4	9.22429	5.119257
SLC35A5	7.91078	17.37549
SLC39A6	7.26005	7.351105
SLC25A1	7.10385	10.06107
SLC37A3	7.06368	29.04458
SLC48A1	6.77426	8.336738
SLC25A29	6.73623	11.61707
SLC23A2	6.58677	6.764668
SLC22A15	6.44345	17.17855
SLC39A9	6.18562	7.195905
SLC31A1	5.99872	7.739965
SLC2A1	5.77547	3.62349
SLC35B1	5.4807	**No data**
SLC35E2B	5.37585	14.90033
SLCO4C1	5.25519	6.557368
SLC46A3	5.17528	5.261813
SLC35E3	4.72873	**No data**
SLC35F5	4.69507	12.34666
SLC26A8	4.56664	3.324936
SLC30A5	4.42129	8.462588
SLC38A1	4.33379	1.82469
SLC19A2	4.02632	0.802967
SLC22A1	3.91727	15.31258
SLC50A1	3.9015	6.878278
SLC35A1	3.80725	**No data**
SLC18A2	3.7171	1.370494
SLC27A3	3.67019	**No data**

SLC25A46	3.57248	4.63701
SLC30A9	3.53037	4.27509
SLC17A5	3.49068	3.028465
SLC25A40	3.45767	8.186168
SLC43A3	3.4212	**No data**
SLC2A14	3.36893	0.906642
FLVCR1	3.3331	11.74628
SLC25A32	3.31725	1.141212
SLC35B3	3.31298	6.968573
SLC12A4	3.29637	**No data**
SLC35E2	3.24559	14.90033
SLC35B2	3.1499	**No data**
SLC25A20	3.0388	13.83024
SLC39A3	3.00139	4.865003
SLC25A16	2.88922	2.370293
SLC37A1	2.87833	**No data**
SLC44A1	2.79355	2.664965
SLC7A6	2.78867	2.361488
SLC39A13	2.78257	4.708685
SLC4A2	2.70562	14.91975
SLC25A45	2.66672	4.06514
SLC30A6	2.55172	3.284525
SLC25A39	2.43246	2.116713
SLC5A9	2.41311	2.3544
SLC25A14	2.27557	4.54853
SLC25A36	2.24725	1.794893
MFSD7	2.17705	4.75029
SLC9A6	2.12071	2.086033
SLC12A1	1.97848	11.74522
SLC6A8	1.96133	2.048953
SLC37A2	1.94582	2.08544
SLC30A7	1.90897	7.608707
SLC25A24	1.90318	2.7849
SLC52A2	1.83537	6.545528
SLC30A1	1.65508	2.871723
SLC35C1	1.6161	2.864028
SLC25A33	1.60194	0.085965
SLC35D1	1.57019	0.861985
SLC26A6	1.53617	3.069608
SLC39A8	1.51151	0.808593

SLC24A3	1.49052	0.976179
SLC6A12	1.48353	3.38105
SLC39A11	1.44917	1.204404
SLC35D2	1.43724	3.107213
SLC25A25	1.39899	1.099743
SLC1A4	1.39617	0.302471
SLC25A13	1.38929	1.150665
SLC16A14	1.34748	0.57943
SLC51A	1.32701	**No data**
SLC25A38	1.32695	1.032691
SLC25A30	1.26993	1.05837
SLC25A22	1.19878	3.907003
SLC5A6	1.17366	1.142233
SLC35A3	1.16899	1.344898
SLC1A5	1.10623	0.933738
MTCH2	1.07897	3.649688
SLC24A4	1.07192	2.226199
SLC25A34	1.04847	1.105145
SLC5A5	1.0317	0.001373
SLC33A1	0.98935	1.418693
SLC5A3	0.967574	**No data**
SLC7A8	0.962858	1.744048
UCP3	0.927209	0.659301
SLC23A1	0.905577	0.376388
SLC4A5	0.891422	0.893974
SLC2A5	0.880448	0.6242
SLC7A11	0.839899	0.126315
SLC12A7	0.816008	0.604336
SLC38A9	0.768943	2.547703
SLC36A2	0.710032	0.017288
SLC25A19	0.679593	0.659763
SLC11A2	0.660908	0.767677
SLC25A15	0.649624	0.352065
SLC27A4	0.64666	1.151295
SLC16A4	0.646018	0.26843
SLC9A7	0.621709	0.838211
SLC26A11	0.601788	0.602508
SLC4A7	0.592421	0.398962
SLC22A5	0.586358	1.134696
SLC10A1	0.582003	0.089458

SLC25A17	0.58162	1.582018
SLC26A2	0.557363	0.367065
SLC37A4	0.545263	1.466712
SLC20A2	0.523111	1.663723
SLC39A4	0.511753	2.772043
SLC15A2	0.494293	**No data**
SLC25A12	0.491284	0.947139
SLC16A13	0.49106	0.266753
SLC9A4	0.472869	0.029917
RHBG	0.454494	0
SLC46A2	0.393315	0.506613
SLC7A1	0.392402	0.113242
SLC41A1	0.39035	0.153188
SLCO4A1	0.386293	0.030919
SLC39A10	0.375107	0.280526
SLC12A2	0.358682	0.251158
SLC10A7	0.354472	0.47415
SLC4A8	0.346928	1.085581
SLC24A1	0.343325	0.556316
SLC16A1	0.341766	0.100955
SLC41A3	0.33136	1.309153
SLC29A3	0.3301	0.613083
SLC28A2	0.319969	0
SLC6A16	0.313818	0.702131
SLC25A53	0.301434	3.752335
SLC25A26	0.300095	0.448138
SLC41A2	0.293223	1.133091
SLC2A6	0.275303	0.812577
SLC2A8	0.273322	0.814157
SLC45A3	0.269156	0.69311
SLC6A20	0.25677	0.06559
SLC18B1	0.256123	0.353239
SLC2A4	0.252583	0.003538
SLC3A1	0.249917	0.161592
SLC6A13	0.235715	0.26222
SLC44A4	0.222833	0.025491
SLC43A1	0.220751	0.140674
SLC25A42	0.217023	0.229577
SLC39A14	0.215165	0.066361
SLC12A8	0.215099	24.8685

SLC2A9	0.213474	1.134367
SLC9A9	0.206143	0.471828
SLC15A1	0.205513	0
SLC27A1	0.190422	0.350563
SLC35F1	0.183551	0
SLC30A4	0.17871	0.043864
SLC25A18	0.173669	0.178288
SLC23A3	0.171536	**No data**
SLC27A2	0.165621	0.147139
SLC17A9	0.163025	0.220222
FLVCR2	0.161085	2.995074
SLC25A35	0.155719	0.474642
SLC25A23	0.152735	0.092535
SLC25A27	0.15039	0.499895
SLC25A10	0.148419	1.767058
SLC2A13	0.142042	0.254337
SLC38A5	0.140477	0.244678
SLC14A2	0.138358	0.023388
SLC35B4	0.135503	0.133596
SLC16A7	0.133516	0.174023
SLC22A13	0.132108	0.031119
SLC22A14	0.128302	0.112629
SLC45A2	0.124561	0.079131
SLC47A1	0.123309	0.295644
SLC14A1	0.120003	0.049875
SLC2A11	0.119388	**No data**
SLC9B2	0.117721	**No data**
SLC25A43	0.110956	0.305292
SLC38A6	0.106089	**No data**
SLC25A2	0.105079	0.012352
SLC28A1	0.105037	0.018301
SLC35E4	0.099345	0.114885
SLC22A17	0.096723	0.019834
SLC7A10	0.096162	0.056653
SLC4A1	0.094702	**No data**
SLC52A1	0.093662	0.002782
SLC7A9	0.091258	0.059758
SLC38A7	0.090202	0.139128
SLC5A10	0.084047	0.008357
SLC16A8	0.076297	0.09536

SLC22A9	0.075539	0
SLC7A14	0.071874	0
SLC35F2	0.07121	0.088443
SLC18A1	0.06938	0.193916
SLC26A4	0.06696	0.013925
SLC35G2	0.066789	0.004921
SLC30A8	0.064359	0.004751
SLC13A4	0.062869	0.292333
SLC9B1	0.06115	0.013052
SLC28A3	0.057827	0.036177
SLC22A16	0.056745	0.14271
SLC32A1	0.055365	0
SLC16A10	0.055337	0.013955
SLC45A1	0.055306	0.026179
SLC24A5	0.055127	0.314604
SLC2A12	0.053854	0.341995
SLC29A2	0.053376	0.038654
SLC25A47	0.049291	0.007801
SLC16A11	0.048024	0
SLC22A23	0.047533	0.146284
SLC25A4	0.047498	0.070657
SLC25A52	0.046697	0.01043
SLC26A1	0.045243	0.203451
SLCO1A2	0.044854	0
SLC4A4	0.044376	0.018906
SLC4A9	0.043369	0.053466
SLC5A11	0.042816	0.009208
SLC6A14	0.040641	0
SLC9A3	0.038749	0.010525
SLC35G1	0.038543	0.138574
SLC1A2	0.038243	0.001444
SLC35G6	0.036646	0
SLC19A3	0.035449	0
SLC27A5	0.03506	0.057876
SLC46A1	0.034287	0.0454
SLC4A10	0.031961	0.010504
SLC29A4	0.031852	0.002375
SLC4A11	0.030189	0.006052
SLC13A1	0.030136	0
SLC13A5	0.028342	0.014783

SLC10A4	0.027983	0.008142
SLC1A3	0.027724	0.080618
SLC16A12	0.027205	0
SLC26A5	0.026607	0
SLC1A7	0.026587	0.053328
SLC35G5	0.026139	0
SLC8A2	0.024413	0.011955
SLC10A5	0.021475	0
SLCO5A1	0.021423	0.01598
SLC9A5	0.017925	0.008151
SLC4A3	0.017676	0.044514
SLC44A3	0.017549	0.089591
SLC38A11	0.017394	0.057391
SLC17A3	0.0168	0
SLC5A2	0.016518	0.188793
SLC34A1	0.014604	0.007438
RHCG	0.014434	0
SLC6A4	0.014124	0.042066
SLC34A3	0.013134	0
SLC6A2	0.012301	0
SLC7A4	0.01204	0
SLC44A5	0.010659	0.004211
SLC24A2	0.009822	0
SLC8A3	0.009751	0.074688
SLC38A3	0.009671	0
SLC36A3	0.009293	0
SLC12A3	0.008825	0.008359
SLC1A1	0.007744	0.014113
SLC12A5	0.006559	0.027903
SLC2A10	0.006494	0.096226
SLC39A5	0.006383	0.008053
SLC16A2	0.006309	0
SLC26A7	0.006272	**No data**
SLC6A3	0.005945	0.001777
SLC22A7	0.005874	0
SLC9A2	0.004401	0.007815
SLC6A9	0.003785	0
SLC13A3	0.002597	0
SLCO1B7	0	0
SLC22A31	0	**No data**

SLC26A10	0	**No data**
SLCO1B1	0	0
SLC25A6	0	17.00073
SLC39A2	0	0.039487
SLC35F3	0	0.032623
SLC35D3	0	0.026302
SLC34A2	0	0.010175
SLC30A2	0	0.009504
SLC27A6	0	0.008934
SLC10A6	0	0.006882
RHAG	0	0.004514
SLC17A7	0	0.004465
SLC22A3	0	0.003755
SLC26A3	0	0.002922
SLC16A9	0	0.002449
SLC52A3	0	0.002154
SLC17A8	0	0.002021
SLCO2B1	0	0.001259
SLC10A2	0	0
SLC13A2	0	0
SLC15A5	0	0
SLC17A1	0	0
SLC17A2	0	0
SLC17A4	0	0
SLC17A6	0	0
SLC18A3	0	0
SLC1A6	0	0
SLC22A10	0	0
SLC22A11	0	0
SLC22A12	0	0
SLC22A2	0	0
SLC22A24	0	0
SLC22A25	0	0
SLC22A6	0	0
SLC22A8	0	0
SLC25A21	0	0
SLC25A31	0	0
SLC25A41	0	0
SLC25A48	0	0
SLC26A9	0	0

SLC2A2	0	0
SLC2A7	0	0
SLC30A10	0	0
SLC30A3	0	0
SLC35F4	0	0
SLC35G3	0	0
SLC38A4	0	0
SLC38A8	0	0
SLC39A12	0	0
SLC47A2	0	0
SLC51B	0	0
SLC5A1	0	0
SLC5A12	0	0
SLC5A4	0	0
SLC5A7	0	0
SLC5A8	0	0
SLC6A1	0	0
SLC6A11	0	0
SLC6A15	0	0
SLC6A17	0	0
SLC6A18	0	0
SLC6A19	0	0
SLC6A5	0	0
SLC6A7	0	0
SLC7A13	0	0
SLC7A2	0	0
SLC7A3	0	0
SLC9C1	0	0
SLC9C2	0	0
SLCO1B3	0	0
SLCO1C1	0	0
SLCO2A1	0	0
SLCO6A1	0	0
UCP1	0	0

The table shows all human SLC transporter genes and their expression values (given as FPKM values) as found in datasets from Wright *et al.* and Chatterjee *et al.*[21,33]. Entries are given as the mean of replicate values, in descending order of expression according to the dataset by Wright *et al.*. Any genes which could not be identified in the dataset by Chatterjee *et al.* are labelled as ‘No data’.

Supplementary Table S4. Expression of ABC transporter proteins is largely consistent across two different datasets.

ABC transporter	Expression (FPKM)	
	**Wright *et al.*, 2013**	**Chatterjee *et al.*, 2016**
TAP1	143.682	456.3108
TAP2	51.0473	132.7189
ABCA7	37.334	178.8746
ABCG1	25.6516	**No data**
ABCA1	17.5845	11.97111
ABCC5	12.5493	16.24293
ABCD1	7.27776	9.552486
ABCF1	7.27122	5.90886
ABCA2	7.15235	18.60513
ABCC1	6.83516	8.063192
ABCF3	4.72307	14.26925
ABCC9	2.21629	0
ABCD3	1.59239	1.14281
ABCF2	1.21202	1.350599
ABCA5	1.18218	1.080757
ABCD4	1.11062	7.700425
ABCB10	1.00666	1.87598
ABCC6	0.970651	2.83314
ABCC10	0.940023	4.91765
ABCB7	0.927628	1.25014
ABCC2	0.839153	17.38025
ABCE1	0.599287	0.6684927
ABCB5	0.53339	0.00458515
ABCB6	0.367747	20.8678
ABCB1	0.319107	0.2100675
ABCB8	0.313178	1.246972
ABCA13	0.291386	0.4219312
ABCC4	0.218874	0.505737
ABCA9	0.139689	0.03220053
ABCB9	0.0938531	0.05274925
ABCB11	0.0664028	0.02136092
ABCC3	0.0612735	2.804117

ABCA3	0.0505832	0.05429485
ABCD2	0.0494921	0.02637948
ABCA10	0.0291777	1.080757
ABCG2	0.026239	0.000778308
ABCB4	0.014383	0.2529438
ABCC11	0.0118731	0.00540443
ABCA6	0.0101404	0.01867935
ABCA4	0.00612525	0.009292483
ABCA12	0	0
ABCA8	0	0
ABCC12	0	0.001889568
ABCC8	0	0
ABCG4	0	0.001287225
ABCG5	0	0
ABCG8	0	0
CFTR	0	0

The table shows all human SLC transporter genes and their expression values (given as FPKM values) as found in datasets from Wright *et al.* and Chatterjee *et al.*[21,33]. Entries are given as the mean of replicate values, in descending order of expression according to the dataset by Wright *et al*.. Any genes which could not be identified in the dataset by Chatterjee *et al.* are labelled as ‘No data’.

Supplementary Figure S5. Zebrafish neutrophils and non-neutrophil cells express distinct subsets of SLC transporter proteins.

a Tree scale: 0.1 **b** Tree scale: 0.1

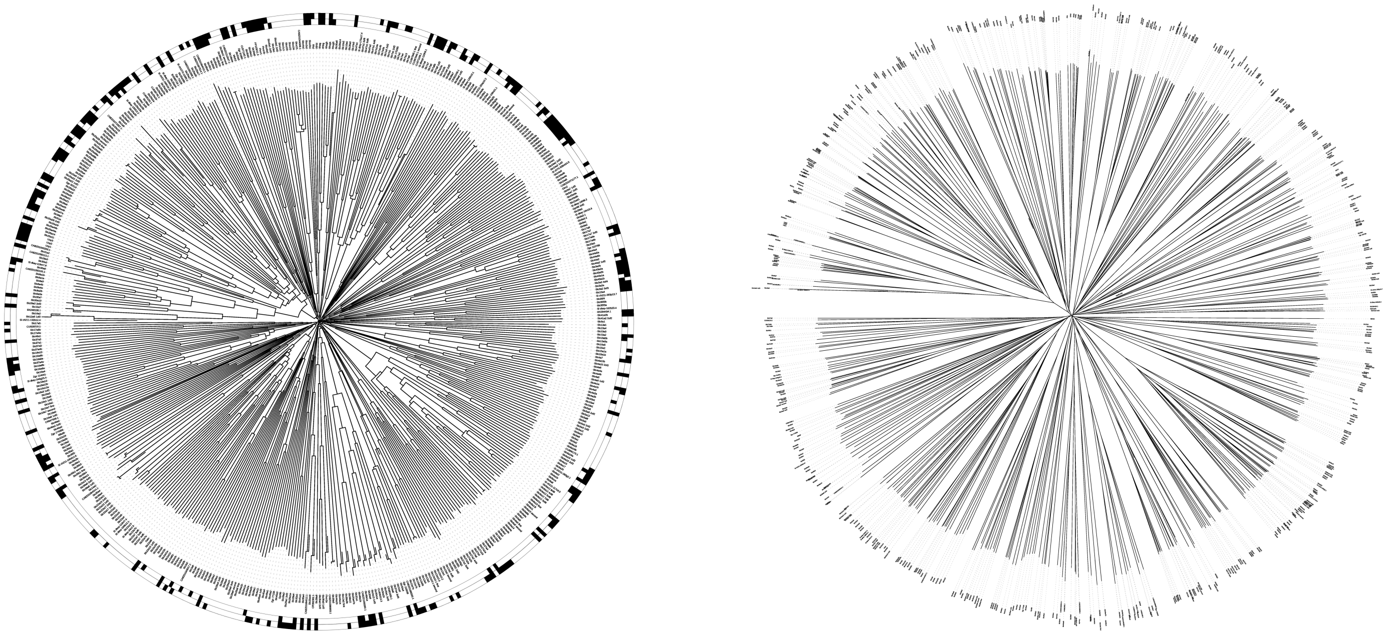

Scalable phylogenetic trees in both (**a**) circular and (**b**) unrooted display modes indicate evolutionary relationships between SLC transporter proteins in zebrafish. Branch lengths are proportional to genetic distance. In (**a**), for both neutrophil (inner circle) and background cell (outer circle) expression data, expressed proteins are marked with a black box, whereas proteins not expressed are unmarked

Supplementary Figure S6. Zebrafish neutrophils and non-neutrophil cells express distinct subsets of ABC transporter proteins.

a

Tree scale: 0.1

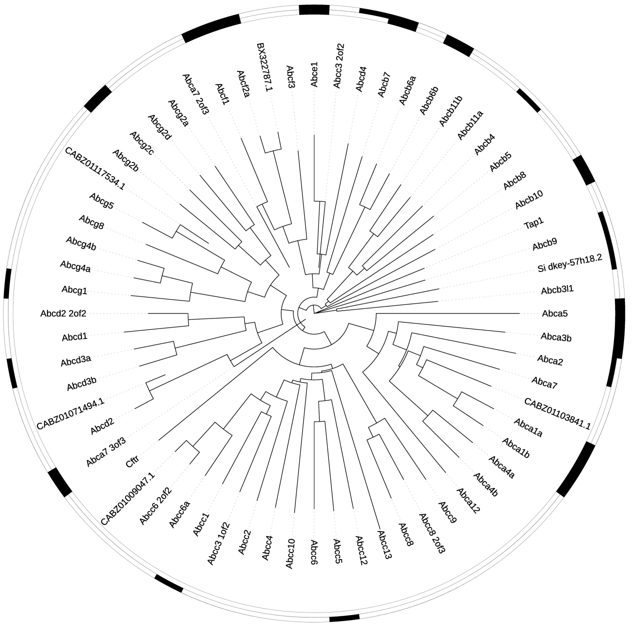

b

Tree scale: 0.1

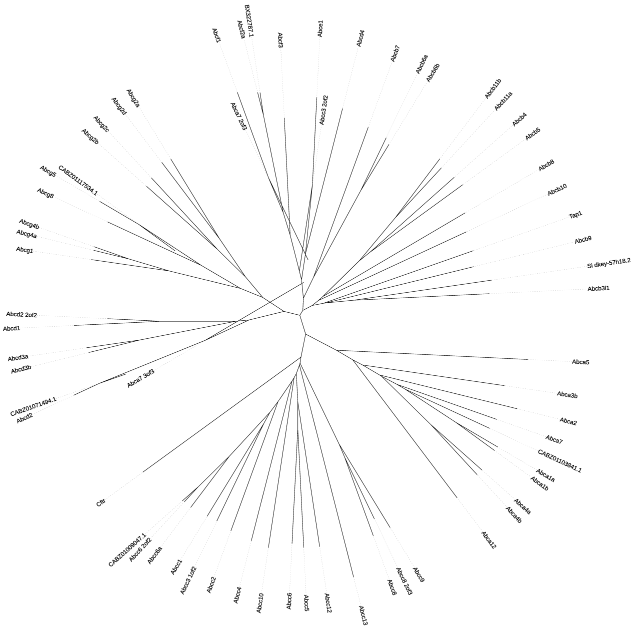

Scalable phylogenetic trees in both (**a**) circular and (**b**) unrooted display modes indicate evolutionary relationships between ABC transporter proteins in zebrafish. Branch lengths are proportional to genetic distance. In (**a**), for both neutrophil (inner circle) and background cell (outer circle) expression data, expressed proteins are marked with a black box, whereas proteins not expressed are unmarked.

Supplementary Table S7. A subset of SLC drug transporters are expressed in both human and zebrafish neutrophils.

Human gene	Zebrafish orthologue gene(s)
DIRC2	*dirc2*
FLVCR1	*flvcr1*
MTCH2	*mtch2*
SLC10A3	*slc10a3*
SLC12A9	*slc12a9*
SLC15A4	*slc15a4*
SLC16A3	*slc16a3*
SLC16A6	*slc16a6b*
SLC19A1	*slc19a1*
SLC1A4	*slc1a4*
SLC20A1	*slc20a1b*
SLC22A15	*slc22a15_1of2*
SLC22A18	*slc22a18*
SLC25A1	*slc25a1a*
SLC25A11	*slc25a11*
SLC25A14	*slc25a14*
SLC25A20	*slc25a20*
SLC25A22	*slc25a22*
SLC25A24	*slc25a24*
SLC25A25	*slc25a25a*
SLC25A28	*slc25a28_first*
SLC25A3	*slc25a3b*
SLC25A32	*slc25a32a*
SLC25A33	*slc25a33*
SLC25A36	*slc25a36a; slc25a36b*
SLC25A39	*slc25a39*
SLC25A40	*slc25a40*
SLC25A44	*slc25a44b*
SLC25A46	*slc25a46*
SLC25A5	*slc25a5*
SLC27A3	*slc27a3*
SLC2A1	*slc2a1b*

SLC2A3	*slc2a3b*
SLC30A1	*slc30a1a*
SLC30A5	*slc30a5*
SLC30A7	*slc30a7*
SLC30A9	*slc30a9*
SLC31A1	*slc31a1*
SLC31A2	*slc31a2*
SLC35A2	*slc35a2*
SLC35C1	*slc35c1*
SLC35D1	*slc35d1a*
SLC35D2	*slc35d2*
SLC35E1	*slc35e1*
SLC35E3	*slc35e3*
SLC36A1	*slc36a1*
SLC37A2	*slc37a2*
SLC38A2	*slc38a2*
SLC39A6	*slc39a6*
SLC39A7	*slc39a7*
SLC3A2	*slc3a2a; slc3a2b;*
SLC40A1	*slc40a1*
SLC43A2	*slc43a2a; slc43a2b*
SLC43A3	*slc43a3b*
SLC44A2	*slc44a2*
SLC48A1	*slc48a1a; slc48a1b*
SLC4A2	*slc4a2b*
SLC51A	*slc51a*
SLC6A6	*slc6a6a*
SLC7A5	*slc7a5*
SLC7A7	*slc7a7*
SLC8B1	*FP103011.3*
SLC9A8	*slc9a8*
SLCO3A1	*slco3a1*
UCP2	*ucp2*

The list shows all SLC drug transporter genes which we found both to be expressed in primary human neutrophils, and which also had at least one corresponding zebrafish orthologue (shown here alongside the relevant human gene) expressed in zebrafish neutrophils.

Supplementary Table S8. A subset of ABC drug transporters are expressed in both human and zebrafish neutrophils.

Human gene	Zebrafish orthologue gene(s)
ABCA1	*abca1a; abca1b*
ABCA2	*abca2*
ABCA5	*abca5*
ABCA7	*abca7_3of3*
ABCB10	*abcb10*
ABCF1	*abcf1*
ABCF2	*abcf2a*
TAP2	*si:dkey-57h18.2*

The list shows all ABC drug transporter genes which we found both to be expressed in primary human neutrophils, and which also had at least one corresponding zebrafish orthologue (shown here alongside the relevant human gene) expressed in zebrafish neutrophils.
